# Ischial tuberosity: new donor site for bone grafts in animal cleft research

**DOI:** 10.1038/s41598-020-77862-w

**Published:** 2020-11-26

**Authors:** Stephan Christian Möhlhenrich, Kristian Kniha, Zuzanna Magnuska, Felix Gremse, Florian Peters, Gholamreza Danesh, Frank Hölzle, Ali Modabber

**Affiliations:** 1 Department of Orthodontics, University of Witten/Herdecke, Alfred-Herrhausen Str. 45, 58455 Witten, Germany; 2 Department of Oral and Maxillofacial Surgery, University Hospital of Aachen, Pauwelsstraße 30, 52074 Aachen, Germany; 3 Institute for Experimental Molecular Imaging, RWTH Aachen University, Forckenbeckstrasse 55, 52074 Aachen, Germany

**Keywords:** Experimental models of disease, Preclinical research

## Abstract

In the context of cleft repair in animal research in rat models, different areas can be used for bone grafting. The aim of the present study was to present the tuberosity of the ischium as a new donor site and to evaluate its quality in relation to an artificial alveolar cleft. Four weeks after creating experimental alveolar clefts in seven Wistar rats, the repair was performed in the now twelve-week-old male animals using bone blocks grafted from the ischial tuberosity. Two days before surgery and two as well as twenty-eight days after surgery, microCT scans were performed, and the grafted bone blocks were analyzed regarding height, width, thickness, and volume. Additionally, bone mineral density (BMD) and bone volume fraction (BV/TV) were measured in the repaired cleft. The mean bone volume of the graft was about 19.77 ± 7.77mm^3^. Immediately after jaw reconstruction the BMD and BV/TV were about 0.54 ± 0.05 g/cm^3^ and 54.9 ± 5.07% for the transplant and about 1.13 ± 0.08 g/cm^3^ and 94.5 ± 3.70%, respectively, for the surrounding bone. Four weeks later the BMD and BV/TV were about 0.57 ± 0.13 g/cm^3^ and 56.60 ± 13.70% for the transplant and about 11.17 ± 0.07 g/cm^3^ and 97.50 ± 2.15%, respectively, for the surrounding bone. A hip fracture was found in four of the animals after surgery. The ischial tuberosity offers large bone blocks, which are sufficient for cleft repair in the rat model. However, the bone quality regarding BMD and BV/TV is less compared with the surrounding bone of the alveolar cleft, even after a period of 4 weeks, despite recognizable renovation processes.

## Introduction

Different rodent models have been established in animal research for the treatment of clefts focusing the bone responses in grafted sites ^1–8^. These alveolar cleft repairs can be performed using various dental materials that include natural bone materials like autografts, allografts or xenografts as well as synthetic bone substitutes such as bioceramics, polymers or biocomposites ^9^.

In cleft treatment, the osteogenic, osteoinductive and osteoconductive properties of autografts make them the gold standard approach in treating bone defects. Furthermore, the autologous graft tissue is already available from different anatomical sites.

The distal and mid-femur as well as the calvaria were introduced in rodent models for testing the efficacy of bone substitute biomaterials ^10^, which were developed to simulate human in vivo environments and physical conditions to test the availability and comparability of bone substitute biomaterials ^11–15^. All these sites can also be seen as possible donor sites for bone grafting. However, the amount of bone is limited, due the fact that compared with larger animals such as rabbits, dogs and pigs, rodents have small-sized long bones and thin and fragile cortices ^16^. Taking into account the possible amount of grafted bone from the respective critical size defect area, the distal femur offers bone of 2 mm in diameter and 2–3 mm in depth ^17,18^, the mid-femur about 5 mm in length ^19,20^ and the calvaria about 8 mm in diameter ^21^.

In general, bone grafts from the hip, especially the iliac crest have proven to be particularly suitable for the reconstruction of the upper jaw ^22^. However, this donor region has not yet been described in the rodent model, even though for the direct comparison with other bone substitutes it would be meaningful. As part of current animal research about the influence of different bone substitutes for jaw reconstruction on subsequent tooth movement, in seven examined rodents autologous bone was grafted from the ischial tuber of the hip. The aim of the present investigation is to provide a detailed description of grafting the non-vascular bone allograft, to assess its quality in terms of quantity and density, and to describe possible postoperative complications.

Furthermore, the bone quality of transplant and surrounding artificial cleft bone were radiological evaluated with regard to bone mineral density (BMD) and bone volume fraction (BV/TV). The first hypothesis of this investigation was that the amount of bone graft is lager compared to other possible donor sites in rats and the second hypothesis supposed that BMD and BV/TV of transplant will be less over the time compared to the surrounding cleft bone according to human bony conditions in cleft patients.

## Materials and methods

Seven 8-week-old male Wistar-HAN rats (Janvier Labs, Le Genest-Saint-Isle, France) with an average weight of 476 ± 31 g served as the experimental animals. The rats demonstrated clinically normal body conditions (body condition score, BCS, ≥ 3.0) ^23^ and belonged to a group within a rodent cleft research project in which the animals were randomly allocated. The a priori sample size calculation was performed using a one-way ANOVA as part of this main research project with regard to root resorption during orthodontic tooth movement in different cleft repairs. The calculation resulted in eight animals per group, including one animal as drop out. Therefore, the present study describes a new method of bone grafting from rats’ hip for cleft repair and no control group existed.

All experiments were conducted in accordance with the German animal welfare law (Tierschutzgesetz, TSchG) and the EU Directive (2010/63/EU). The study protocol was approved by the Governmental Animal Care and Use Committee (Reference No.: 81-02.04.2018.A342; Landesamt für Natur, Umwelt und Verbraucherschutz Recklinghausen, Fachbereich 81, Nordrhein-Westfalen, Germany; dated: 14.02.2018). The study protocol complied to the ARRIVE Guidelines ^24^ and with the Guide for the Care and Use of Laboratory Animals. All animals were group-housed in filter-top cages (Type 2000, Tecniplast, Buguggiate, Italy) and for cage enrichment, low-dust wood granulate was used as bedding (Rettenmeier Holding AG, Wilburgstetten, Germany) in addition to nesting material (Nestlet, 14010, Plexx B.V., Elst, The Netherlands).

After surgery, the rats were given special soft food (DietGel Boost, Clear H2O, Portland, USA) for 7 days followed by standard diet (rat/mouse maintenance #V1534-300, 10 mm; ssniff Spezialdiäten GmbH, Soest, Germany) and water ad libitum. None of the animals in this group must be excluded during the experiment due a worsened state of health. Human end points were defined as body weight decrease ≤ 20%, BCS ≤ 2, signs of severe disturbed circulation, self-mutilation or uncoordinated behavior.

### Surgical procedure

Four weeks after creating an experimental alveolar cleft in the left side of the rats’ upper jaws, the cleft repair using autologous bone grafts from the hip was performed in now 12-week-old male Wistar rats with an average weight of 510 ± 28 g. Preoperatively the rats were anesthetized with an intraperitoneal injection of a combination of ketamine (80–100 mg/kg; Ketavet, Pfizer, Berlin, Germany) and medetomidine (0.15–0.25 mg/kg, Domitor, Orion Pharma, Espoo, Finland). Additionally, endotracheal intubation using a 15-gauge intravenous catheter was performed to substitute oxygen. Buprenorfine (0.03–0.05 mg/kg Temgesic, Indivior Limited, Berkshire, UK) was administered subcutaneously as a pain killer at 8-h intervals, and cefuroxime (15 mg/kg s.c.; Fresenius, Bad Homburg, Germany) as antibiotic was started at 24-h intervals. Both were administered for 7 days with regard to cleft repair and post-operative pain. Immediately after surgery atipamezole hydrochloride (0.75 mkg/kg, Antisedan, Orion Pharma, Espoo, Finland) was given as a reversing agent.

**Figure 1:**
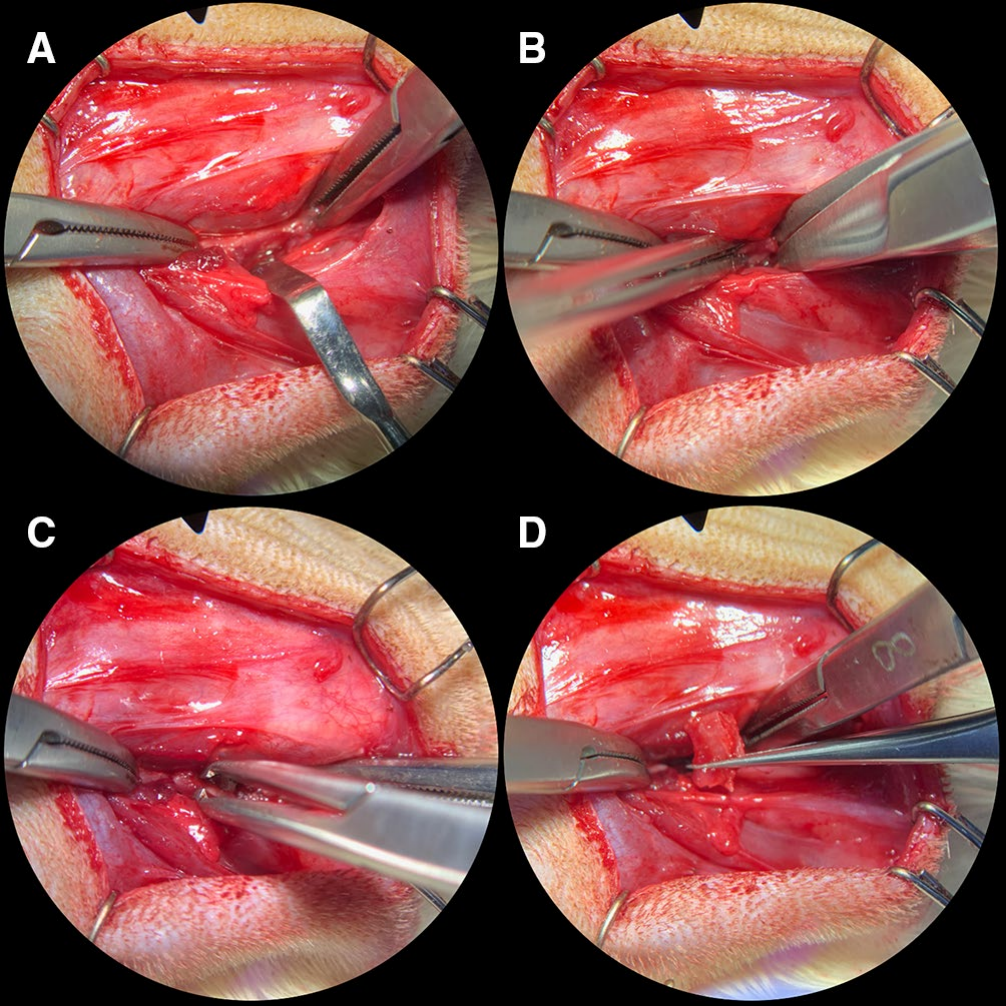
Bone harvesting: Surgical procedure for harvesting cortical bone blocks from the rat pelvis: (**A**) Approach to the ischial tuber of the hip by means of a 10-mm long incision paramedian to the spine, skin and muscle mobilization and finally bone fixation by two bone forceps (both lateral), (**B**) 5–6-mm depth vertical bone cuts using curved dissecting scissors along the bone forceps, (**C**) Bone block mobilization by breaking the tuberosity of the ischium at its thinnest part between the two vertical bone cuts using a micro bone grasping forceps, (**D**) the grafted bone block.

**Figure 2:**
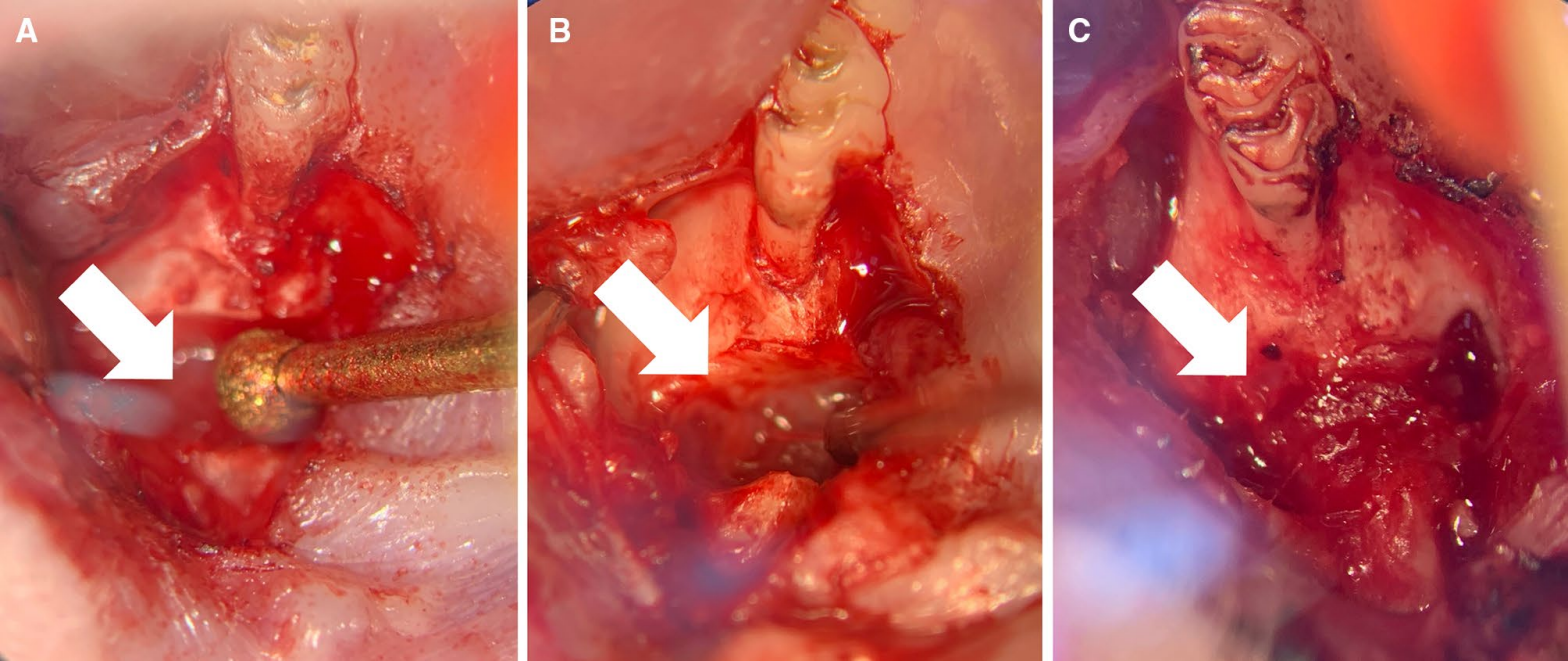
View of the surgical site on the left maxilla and palate: tongue base above, mouth tip below. Preparation of the artificial alveolar cleft (arrow) in the front of the first molar on the left side of rats' maxilla using an ultrasonic device (**A**) before (**B**) and after (**C**) cleft repair with autologous bone graft from the hip.

The animals were placed in prone position and after shaving and disinfection of the wound area, an approximately 10-mm-long incision was made caudal to the greater trochanter, approximately 5 mm lateral and parallel to the spine. Afterwards the preparation was carried out deeply until the superficial muscles were able to be observed and the central part of the gluteus maximus and gluteus medius muscle were mobilized. The superficial attachment of the gluteus minimus muscle was released caudal towards the ischial tuber so that between muscle structures an access to the hip bone was created (Fig. 1A). For bone block removal from the hip’s ischial tuber, two 5–6 mm deep vertical bone cuts with a distance ranging between 5 and 10 mm were performed using a curved dissecting scissor (Fig. 1B). Subsequently, the bone was mobilized and removed using micro bone grasping forceps (Fig. 1C, D). After the non-vascular bone graft was lifted, the muscles were sewn using single interrupted stitches with 5–0 vicryl sutures and the reflected skin in a single layer using 5–0 nylon sutures. Finally, the bone block was prepared and used for upper jaw reconstruction (Fig. 2A, B).

### Cleft creation and repair

The clefts defects were created in 8-week-old rats by a micrometric osteotomy between the roots of the incisor and the first molar using an ultrasonic device with special insert with a diameter of 1.7 mm (OT5, Mectron s.p.a., Carasco, Italy) (Fig. 2A). Following that bone wax (Bonewax, Ethicon, Johnson & Johnson Medical GmbH, Norderstedt, Germany) was applied to maintain the artificial alveolar cleft and the wound was closed by resorbable sutures (7/0 Vicryl, Ethicon, Johnson & Johnson Medical, Somerville, NJ. US).

Before cleft repair the bone wax was removed and the surrounding bone of the cleft was refreshed (Fig. 2B). Afterwards, the grafted bone block was prepared and used for upper jaw reconstruction by a combination of press fit and layer technique (Fig. 2C).

### Microfocus computed tomography (microCT)

Two days before and two as well as twenty-eight days after surgery, imaging using in-vivo microCT system (U-CT OI, MILabs, Utrecht, the Netherlands) of the rats was done for radiographic analysis of the skeletal anatomy to assess the feasibility of grafting bone blocks from the hip under general anaesthesia using isoflurane [induction: 5 vol% isoflurane + 5 L O_2_/min; maintenance: 2 vol% isoflurane + 2 L O_2_/min] (Abbott GmbH & Co. KG, Wiesbaden, Germany). After positioning in a rat bed, the hip of a rat was scanned with an ultra-focus magnification through 360° of rotation at a 0.75° increment with 0.3 s/degree. The microCT data was reconstructed at an isotropic voxel size of 40 µm. For analysis, the microCT data was down-sampled using binning to a voxel size 80 µm. Images were evaluated using cross-sectional slices and rendered three-dimensional iso-surfaces.

**Figure 3:**
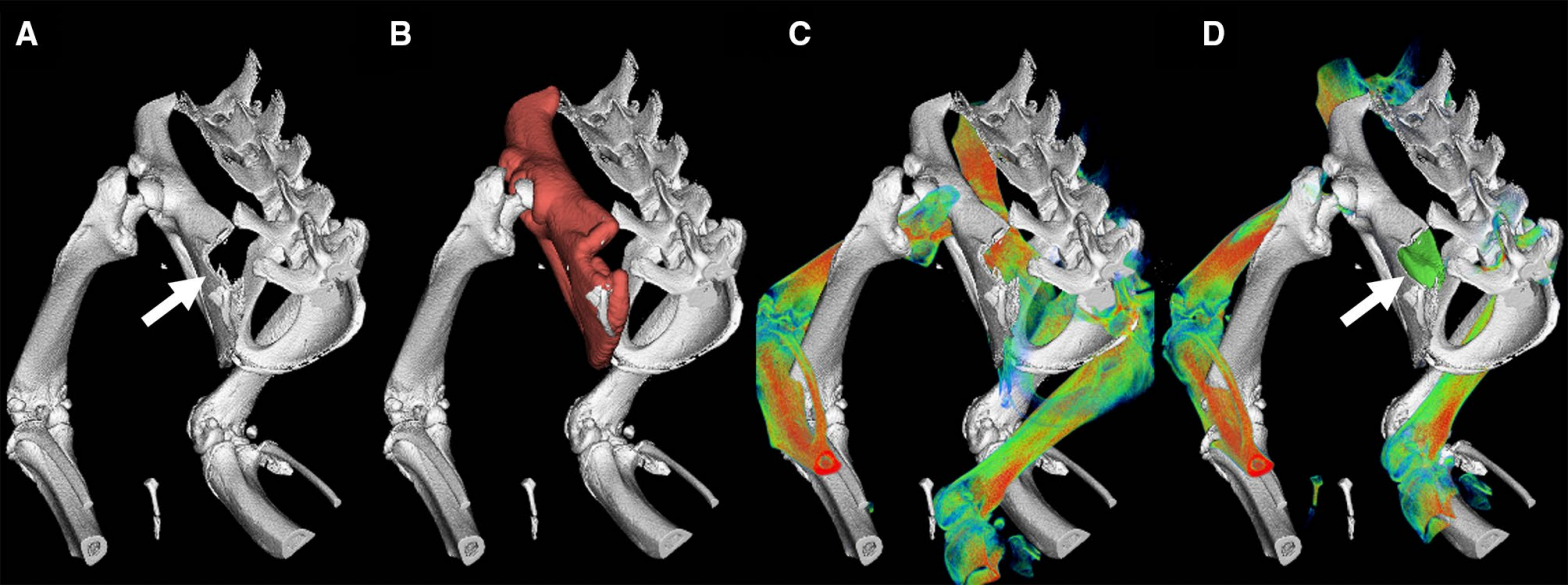
Image fusion to assess bone defect size to determine the size and volume of the grafted bone block: (**A**) microCT scan after surgery with the bone defect (arrow) loaded as underlay, (**B**) Segmentation of the left hip using thresholding, cutting and region growing, (**C**) Scan before surgery without bone defect loaded as overlay, (**D**) Alignment and fusion module of the overlay and underlay scan, restricted to the segmented left hip to segment the missing bone block (arrow). Figures were generated with Imalytics Preclinical software 2.1 (Gremse-IT, Aachen, Germany) ^25^.

To assess the bone defect size, the pre- and postoperative images were fused (Fig. 3A–D). Therefore, the microCT scan after surgery was loaded as underlay, and the left hip was segmented using thresholding, cutting and region growing ^25^. Afterwards, the scan before surgery was loaded for segmentation, and the corresponding overlay was aligned to the underlay by a fusion module restricted to the segmented left hip. In this way, the grafted bone block was segmented and analyzed in terms of height, width, thickness, and volume.

**Figure 4:**
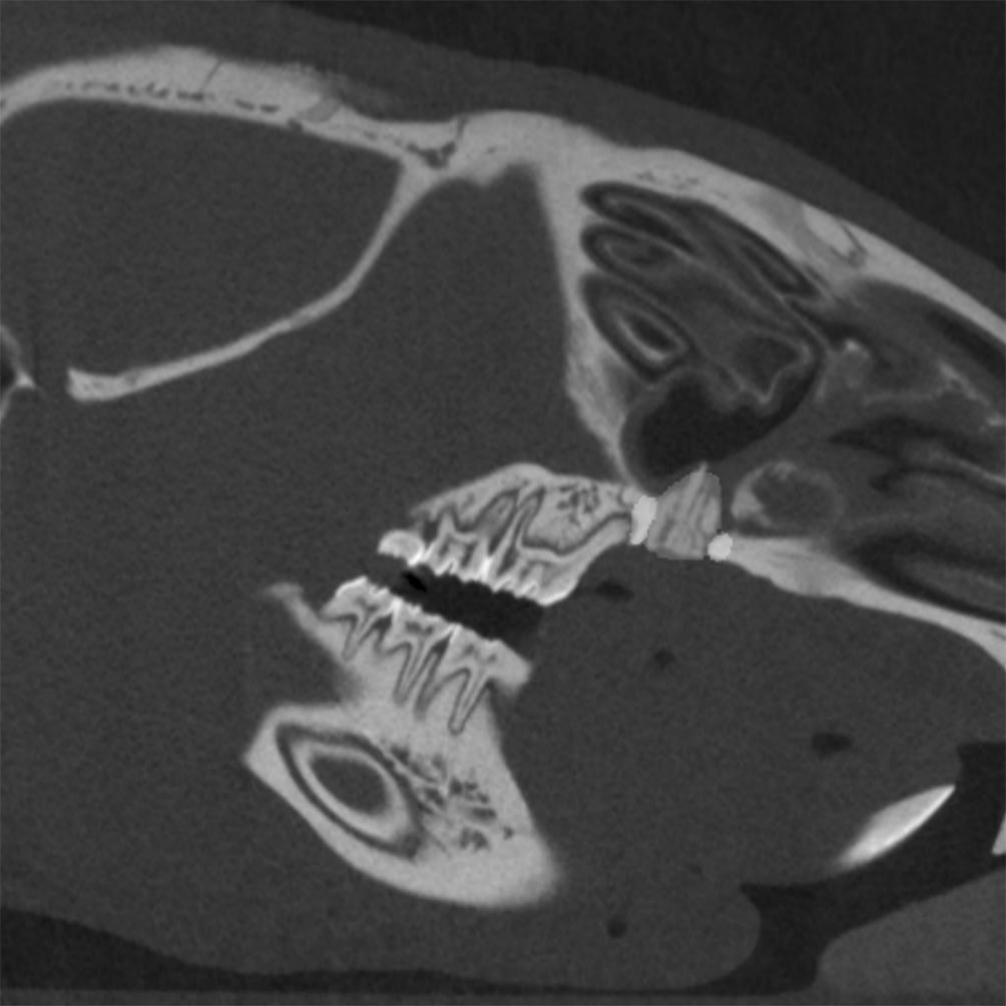
Sagittal view of a CT scan after cleft repair for analyzing the bone quality of the augmented bone into the cleft (dark grey/green area) and the surrounding alveolar bone (light grey/beige area).

Furthermore, the bone graft as well as the surrounding bone of the cleft area was investigated concerning mineral density (BMD in g/cm3) and volume fraction (BV/TV in %) 2 and 28 days after cleft repair within the context of the main experimental research. To define the surrounding bone of the cleft area, a coating of fixed thickness (in our case 10 voxels) was computed using morphological dilation around previously segmented bone graft. Then, the hard tissue was segmented within the coating volume by thresholding ^26^ (Fig. 4).

### Statistical analysis

Descriptive data were collected, and the Kolmogorov–Smirnov test was and normal distribution was found. However, due to the small sample size, the Mann–Whitney test was employed to analyze differences between the bone for reconstruction and the local surrounding bone of the cleft regarding the outcome parameters BMD and BV/TV. The Wilcoxon matched-pairs signed rank test was used for comparing each outcome between the second and twenty-eighth day after cleft repair. The level of significance was set at *P* ≤ 0.05 using the statistical program Prism (version 8, GraphPad Software Inc., La Jolla, CA, USA). All results are expressed as mean ± standard deviation values.

### Ethical approval

The experimental animal study protocol was approved by the Governmental Animal Care and Use Committee (Reference No.: 81-02.04.2018.A342; Landesamt für Natur, Umwelt und Verbraucherschutz Recklinghausen, Nordrhein-Westfalen, Germany; dated: 14.02.2018). The study protocol conforms to the ARRIVE Guidelines and with the Guide for the Care and Use of Laboratory Animals. All applicable international, national, and/or institutional guidelines for the care and use of animals were followed.

### Informed consent

For this type of study, formal consent is not required.

## Results

All animals were in good oral and physical health, and the healing of the soft tissue was completed 7 to 10 days after bone harvesting. Also, none of these animals had to be euthanized during the whole experimental period. Additionally, no exposure of the grafted bones or signs of inflammation were observed.

The average size of the grafted bone blocks was in length about 7.41 ± 1.47 mm and height about 4.37 ± 1.72 mm. The blocks demonstrated a mean thickness of 0.87 ± 0.26 mm and a mean bone volume about approximately 19.77 ± 7.77 mm^3^ that ranged between 11.10 and 30.77 mm^3^.

**Figure 5:**
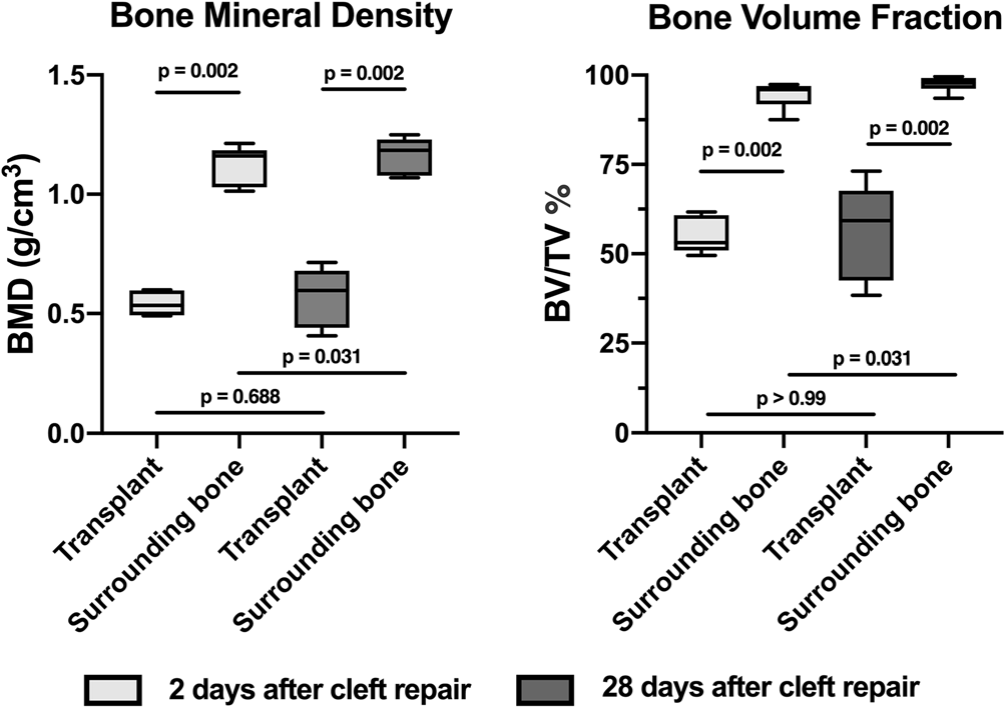
Boxplots of the bone mineral density (BMD) and bone volume fraction (BV/TV) of the transplant and the surrounding bone to the alveolar cleft 2 and 28 days after cleft repair with the *P* values of the corresponding comparisons. Figures were generated with Imalytics Preclinical software 2.1 (Gremse-IT, Aachen, Germany) ^25^.

Figure 5 depicts boxplots of the BMD and BV/TV of the transplant and the bone surrounding the alveolar cleft as well as the *P* values of the corresponding comparisons.

**Figure 6:**
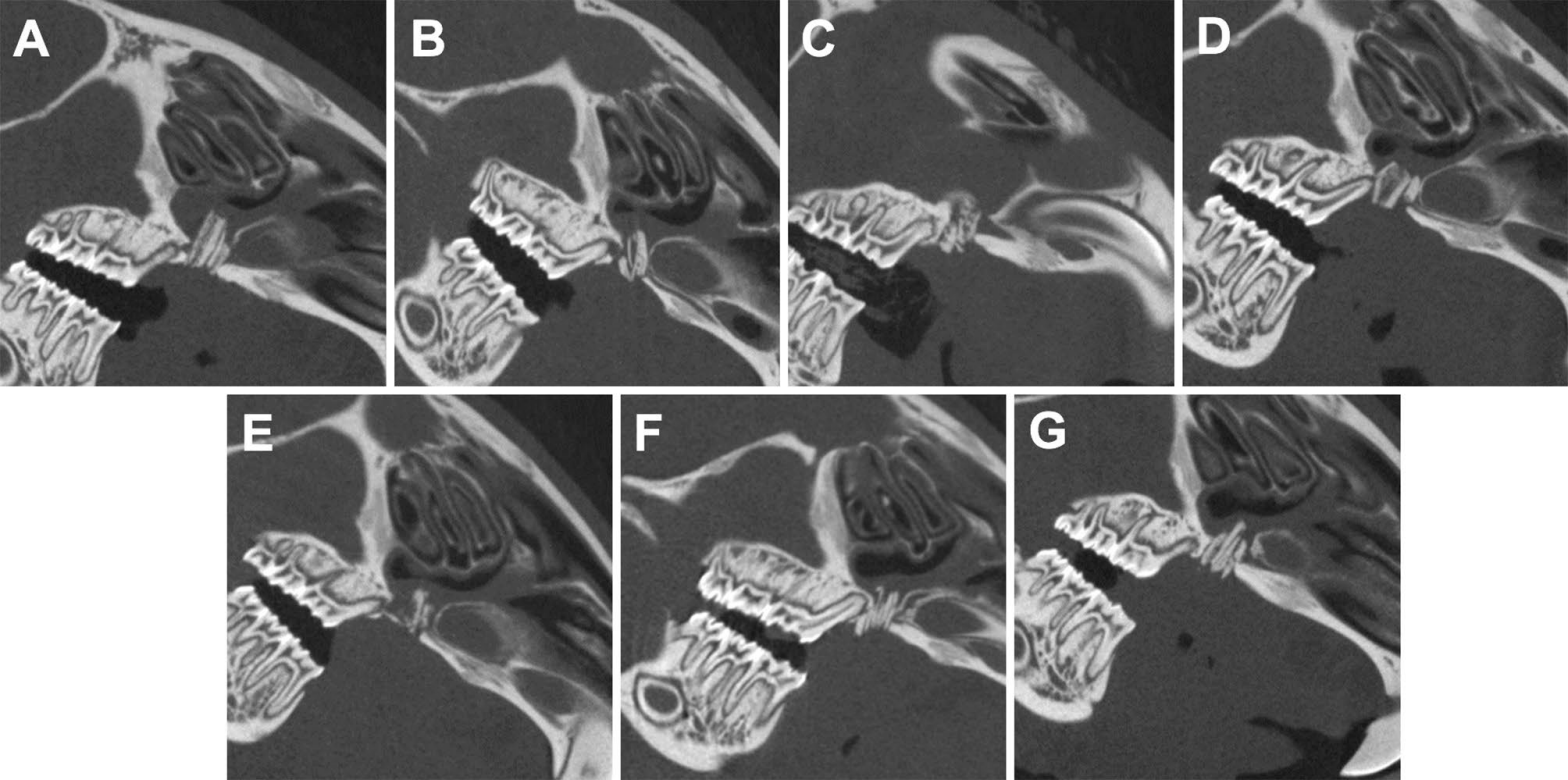
Sagittal view of all CT scans two days after cleft repair demonstrating an over contouring into the nasal cavity of varying degrees. Figures were generated with Imalytics Preclinical software 2.1 (Gremse-IT, Aache, Germany) ^25^.

Nearly all jaw reconstruction demonstrated over contouring into the nasal cavity of varying degrees (Fig. 6). However, the animals showed no further associated restrictions in the postoperative phase. Immediately after jaw reconstruction, the BMD of the transplant was approximately 0.54 ± 0.05 g/cm^3^, and for the surrounding bone, it was approximately 1.13 ± 0.08 g/cm^3^. The corresponding mean values for BV/TV of the transplant and surrounding bone were 54.9 ± 5.07% and 94.5 ± 3.70%, respectively. Four weeks after cleft repair, the BMD of the transplant was nearly 0.57 ± 0.13 g/cm^3^, and for the surrounding bone, it was approximately 1.17 ± 0.07 g/cm^3^. The corresponding mean values for BV/TV of the transplant and surrounding bone were 56.60 ± 13.70% and 97.50 ± 2.15%, respectively. Significant differences regarding the bone qualities (BMD and BV/TV) were found for all comparisons between the bone of the transplant and the surrounding bone of the cleft (*P* = 0.022), as well between immediately after surgery and 28 days after surgery for BMD (*P* = 0.031). Furthermore, in the postoperative CT scan, a hip fracture was discovered in four animals (57%), but these animals were not impaired over the further test period. The fractures occurred unexpectedly, which is why they were not defined as an abort criterion in advance.

## Discussion

Cleft repair can be done by different types of autologous bone grafts (e.g., iliac crest, cranium, tibia, rib, and mandibular symphysis) as well as as well as tissue-engineered materials like allografts or xenografts, and synthetic bone substitutes ^9,27–29^. However, bone grafts from the iliac crest have proven to be particularly suitable and were considered the gold standard for cleft repair due to the osteogenic, osteoinductive, and osteoconductive properties ^22^. Nevertheless, efforts continue to improve surgical techniques and bone substitutes to enhance the clinical outcome in order to avoid complaints associated with autologous bone grafts from the iliac crest, which include postoperative pain, gait disturbances as well as chronic sufferings like iliac contour alterations, unsightly scars and recurring discomfort ^30^.

In corresponding animal research, various rat models have been described as focusing the bone responses at recipient sites ^1–3,5–8,31,32^ and different areas have been established to enable critical-size bone defects in rat models ^10^. These regions can also be viewed as potential donor sites for grafting non-vascular allografts in rats, which could be of interest in cases of jaw reconstruction. Due to the specific anatomy of rodents, the iliac crest, which is the common donor site for bone grafting in human for cleft repair is difficult to access, which probably suggests less available bone. In this context, Sun et al. reported on the use of bone from the iliac crest of rats for jaw reconstruction but did not explain the procedure. By contrast, the tuberosity of the ischium is more pronounced and easier to approach in rats. To the best of our knowledge, there are currently no reports about this region as a possible donor side in cleft research in rats. Therefore, the aim of the present exploration as a sub-project of an investigation on different types of osteoplasty during cleft repair was to describe this donor side for the first time and to consider the possible amount of bone as well as its density (BMD, BV/TV) compared to the surrounding bone of the alveolar cleft.

The surgical approach to the tuberosity of the ischium in the rats was simple since the palpation allowed a good indication of the donor side location. Regional muscles only had to be slightly damaged because they could be raised and moved sideways. Only the superficial attachment of the gluteus minimus muscle had to be cut in the area of the donor region to release it caudally the ischial tuber. Deep muscle sutures were therefore hardly necessary. In principle, the bone block can also be removed easily. Here, it is recommended that the desired area be fixed with two bone forceps. Due to the relatively thin bone structure, the bone can easily be cut with a curved dissecting scissor and then broken out with micro bone grasping forceps. However, it must be ensured that the lower bone cut runs above the dorsal margin of the tuberosity of the ischium. Otherwise, a pelvic fracture is possible. All fractures that were found postoperatively in the CT scan were among those animals that were operated first without following this recommendation and which had already been noticed when the lower vertical bone cut was performed. Due to the low bleeding tendency, neither the use of bone wax nor electric cautery was necessary.

Regarding the amount of bone and its quality, it can be assumed that these are likely to be comparable or possibly even more profitable. However, a comparison of the potential bone quantity and quality in this context is difficult because this has not been investigated to date, especially regarding the possible amount of harvested bone. Therefore, conclusions can only be drawn indirectly based on the specified defect sizes. Regarding the distal femur, Kondo et al. reported on defect sizes that were 2 mm in diameter and depth, and Tielinen et al. reported on defect sizes that were 2 mm in diameter and 3 mm in length ^17,18^. Larger defects have been described for the mid-femur site. Both Ohgushi et al. and Kirker-Head et al. reported defect sizes of 5 mm in length ^19,20^. The largest possible amount of bone supply was reported by Yoon et al., who described critical size defects in rat calvaria nearly 8 mm in diameter ^21^. Unfortunately, there is no exact information relating to defect size in all three-room levels; therefore, no statement can be made about the amount of bone removed. However, assuming that the reported values represent the average largest length of the defects and comparing these with the results of the present investigation, it can be concluded that the tuberosity of the ischium with an average size of 4.37 × 0.87 × 7.4 mm and a volume of 19.77 ± 7.77 mm^3^ offers more bone for grafting. Thus, the first hypothesis could be confirmed. However, it should be borne in mind that some animals have suffered fractures of varying degrees, even they did not appear to be clinically impaired in the postoperative phase. These fractures are presumably due to the establishment process of a new bone grafting procedure as well as the amount of bone removal. Therefore, only the amount of bone that actually really required should grafted to keep the risk of fracture as low as possible.

The cortical bone density of the human maxilla range approximately between 810 and 940 Hounsfield units (HU) at the alveolar bone and between 835 and 1113 HU at the basal cortical bone ^33^. In this context, Moscarino et al. reported about an even higher bone density in alveolar cleft patients compared to non-cleft patients in the defect region ^34^. With regard to the BMD after jaw reconstruction with secondary bone graft from the iliac crest, Zhang et al. found a BMD after 3 months about 406.51 HU and after 6 months about 409.53 HU ^35^. They concluded that the alveolar density remained stable at least in this timeslot, whereas bone height level declined during that period and that the latter indicates bone graft absorption over time.

This current research study in rats shows a similar ration to these findings. It was also found that the used bone for reconstruction demonstrated statistically significant BMD and BV/TV compared to the recipient region shortly after insertion and after 4 weeks. Therefore, the second hypothesis was also confirmed. However, there was a slight increase in both parameters but without statistical significance for the bone graft from 2 to 28 day, probably as a result of osseous integration and resorptions that might suggest a corresponding healing process. However, this can only be confirmed histologically and unfortunately, this information was not available due the present findings were parts of subproject. In this context, Sun et al. reported on new bone formation in the cleft area of rats ^2^. However, they reported that no obvious osteoblasts and osteoclasts existed and thus basically halted bone remodelling.

The interpretation of the results is limited by the age of the used animals. In the literature, the postnatal maturity for rats is reported in mean starting at day 49 as peri adolescent and at day 70 as young adulthood. In the present study the cleft was created around day 56. Thus, the animals were in the pubertal age according to Sengupta ^36^. However, the cleft repair took place around postnatal day 84. At this point in time, the animals are already in the adolescent phase. In this context, Brudnicki et al. reported that secondary alveolar bone graft performed before 8 years of age can have limited negative effect on craniofacial morphology ^37^. But with regard to the donor site alveolar bone grafting at an earlier age does not increase donor site symptoms, surgical duration or hospitalization following surgery ^30^.

Furthermore, it must be taken into account that this animal model based on an artificially created cleft while in clinical practice the cleft is innate. However, in context of the actual tendency to perform bone grafting and alveolar cleft repair at an earlier age in the surgical treatment protocol and with regard to the associated potentially higher risk of donor site-related complaints or surgical duration in younger patients ^38–40^, the current model may be ideal for investigation the age-related correlations of bone grafting results.

## Conclusion

The present investigation reported for the first time on non-vascular allografts from the tuberosity of the ischium of the pelvis in rats. The amount of bone appeared superior compared to other potential donor sites, such as the femur or calvaria. The density was less compared to the surrounding bone of an alveolar cleft, however, it suggested bone remodelling over a period of 4 weeks.

## References

[1] Mostafa NZ (2014). Reliable critical sized defect rodent model for cleft palate research. J. Craniomaxillofac. Surg..

[2] Sun J (2017). Biological Effects of orthodontic tooth movement into the grafted alveolar cleft. J. Oral Maxillofac. Surg..

[3] Sun J, Xu Y, Chen Z (2015). Establishment of a rat model for alveolar cleft with bone wax. J. Oral Maxillofac. Surg..

[4] Ru N (2013). In vivo microcomputed tomography evaluation of rat alveolar bone and root resorption during orthodontic tooth movement. Angle Orthod..

[5] Ru N (2016). Microarchitecture and biomechanical evaluation of boneceramic grafted alveolar defects during tooth movement in rat. Cleft Palate Craniofac. J..

[6] Ru N (2016). BoneCeramic graft regenerates alveolar defects but slows orthodontic tooth movement with less root resorption. Am. J. Orthod. Dentofac. Orthop..

[7] Nguyen PD (2009). Establishment of a critical-sized alveolar defect in the rat: a model for human gingivoperiosteoplasty. Plast. Reconstr. Surg..

[8] Jahanbin A (2016). Success of maxillary alveolar defect repair in rats using osteoblast-differentiated human deciduous dental pulp stem cells. J. Oral Maxillofac. Surg..

[9] Sharif F (2016). Dental materials for cleft palate repair. Mater Sci. Eng. C Mater Biol. Appl..

[10] Li Y (2015). Bone defect animal models for testing efficacy of bone substitute biomaterials. J. Orthop. Translat..

[11] Zwingenberger S (2013). Establishment of a femoral critical-size bone defect model in immunodeficient mice. J. Surg. Res..

[12] Zanchetta P (2012). Mixture of hyaluronic acid, chondroitin 6 sulphate and dermatan sulphate used to completely regenerate bone in rat critical size defect model. J. Craniomaxillofac. Surg..

[13] Skaliczki G (2012). Compromised bone healing following spacer removal in a rat femoral defect model. Acta Physiol. Hung..

[14] Kumar S, Ponnazhagan S (2012). Mobilization of bone marrow mesenchymal stem cells in vivo augments bone healing in a mouse model of segmental bone defect. Bone.

[15] Bateman JP (2012). Exploratory study on the effect of osteoactivin on bone formation in the rat critical-size calvarial defect model. J. Periodontal. Res..

[16] Rodeo SA (2017). Translational animal models in orthopaedic research. Am. J. Sports Med..

[17] Kondo N (2005). Bone formation and resorption of highly purified beta-tricalcium phosphate in the rat femoral condyle. Biomaterials.

[18] Tielinen L (2001). Inability of transforming growth factor-beta 1, combined with a bioabsorbable polymer paste, to promote healing of bone defects in the rat distal femur. Arch. Orthop. Trauma Surg..

[19] Ohgushi H, Goldberg VM, Caplan AI (1989). Repair of bone defects with marrow cells and porous ceramic Experiments in rats. Acta Orthop. Scand..

[20] Kirker-Head C (2007). BMP-silk composite matrices heal critically sized femoral defects. Bone.

[21] Yoon E (2007). In vivo osteogenic potential of human adipose-derived stem cells/poly lactide-co-glycolic acid constructs for bone regeneration in a rat critical-sized calvarial defect model. Tissue Eng..

[22] Canady JW (1993). Suitability of the iliac crest as a site for harvest of autogenous bone grafts. Cleft Palate Craniofac. J..

[23] Ullman-Cullere MH, Foltz CJ (1999). Body condition scoring: a rapid and accurate method for assessing health status in mice. Lab. Anim. Sci..

[24] Kilkenny C (2010). Animal research: reporting in vivo experiments: the ARRIVE guidelines. Br. J. Pharmacol..

[25] Gremse F (2016). Imalytics preclinical: interactive analysis of biomedical volume data. Theranostics.

[26] Becker K (2019). Microstructural volumetric analysis of lateral ridge augmentation using differently conditioned tooth roots. Clin. Oral. Investig..

[27] Bajaj AK, Wongworawat AA, Punjabi A (2003). Management of alveolar clefts. J. Craniofac. Surg..

[28] Aalami OO (2004). Applications of a mouse model of calvarial healing: differences in regenerative abilities of juveniles and adults. Plast. Reconstr. Surg..

[29] Kamal M (2018). Volumetric comparison of autogenous bone and tissue-engineered bone replacement materials in alveolar cleft repair: a systematic review and meta-analysis. Br. J. Oral. Maxillofac. Surg..

[30] Brudnicki A (2019). Secondary alveolar bone grafting in cleft lip and palate: a comparative analysis of donor site morbidity in different age groups. J. Craniomaxillofac. Surg..

[31] Mehrara BJ (2000). A rat model of gingivoperiosteoplasty. J. Craniofac. Surg..

[32] Cheng N (2017). Effects of bisphosphonate administration on cleft bone graft in a rat model. Cleft Palate Craniofac. J..

[33] Park HS (2008). Density of the alveolar and basal bones of the maxilla and the mandible. Am. J. Orthod. Dentofacial. Orthop..

[34] Moscarino S (2019). Bone and soft tissue palatal morphology and potential anchorage sides in cleft palate patients. Ann. Anat..

[35] Zhang DZ (2015). Evaluation of bone height and bone mineral density using cone beam computed tomography after secondary bone graft in alveolar cleft. J. Craniofac. Surg..

[36] Sengupta P (2013). The laboratory rat: relating its age with human's. Int. J. Prev. Med..

[37] Brudnicki A (2020). Effects of different timing of alveolar bone graft on craniofacial morphology in unilateral cleft lip and palate. Cleft Palate Craniofac. J..

[38] Precious DS (2009). A new reliable method for alveolar bone grafting at about 6 years of age. J. Oral. Maxillofac. Surg..

[39] Brudnicki A, Brudnicka R, Sawicka E (2014). Outcome of alveolar bone grafting in patients with unilateral cleft lip and palate operated by one-stage method. Dev. Period. Med..

[40] Dissaux C (2016). Evaluation of success of alveolar cleft bone graft performed at 5 years versus 10 years of age. J. Craniomaxillofac. Surg..

